# Examining complex academic language in children with dyslexia: a comparative analysis with curriculum standards

**DOI:** 10.1007/s11881-025-00357-8

**Published:** 2026-02-17

**Authors:** Douglas B. Petersen, Gwendolyn H. Stout, Alisa Konishi-Therkildsen, Norma Hancock, Camryn Lettich, Tiffany Hogan

**Affiliations:** 1 Department of Communication Sciences and Disorders, Baylor University, One Bear Place #97332, Waco, 76798 TX United States; 2 Baylor University, Waco, United States; 3 University of Alaska Anchorage, Anchorage, United States; 4 MGH Institute of Health Professions, Boston, United States

## Abstract

While approximately 50% of children with dyslexia are reported to have Developmental Language Disorder (DLD), there is a possibility that a larger proportion exhibit subclinical weaknesses in complex academic language that detrimentally impact reading comprehension. This study aimed to evaluate the comprehension and production of complex academic language among school-age students with dyslexia who do and do not have DLD across discourse, sentence, and vocabulary levels, comparing their performance against established curriculum standards. Narrative retell language samples were collected from 183 students with dyslexia with and without DLD spanning first to sixth grade. We used the Narrative Language Measures-Listening subtest from the CUBED-3 to assess discourse complexity, sentence complexity, and vocabulary complexity. The findings revealed significant language deficiencies across all grades. Specifically, the majority of students failed to meet benchmark expectations for the inclusion of discourse elements, 94% to 100% of students across grade levels lacked complex sentence structures, and 82% to 100% did not incorporate complex vocabulary as expected by curriculum standards. Notably, 100% of fourth and sixth graders who had dyslexia were found to be below the expected standards in narrative discourse, sentence, and vocabulary complexity. Results also indicated that there was no significant difference between the children with dyslexia who had DLD and those who did not on discourse complexity across most grades. Minimal differences in sentence complexity and vocabulary complexity were observed between the children with dyslexia who had DLD and those who did not. When examining the percent of students with dyslexia who did not have language disorder performing below benchmark expectations, results indicated that all students except for a few first graders were below benchmark. Conclusion The high incidence of language weaknesses observed suggests that beyond the commonly recognized challenges with dyslexia, these students often struggle significantly with language skills that are critical for academic success. Therefore, systematic oral language screening and targeted language interventions are crucial in supporting the broader educational needs of children with dyslexia.

Dyslexia is a neurodevelopmental disorder that affects approximately 7–10% of the population (Shaywitz et al., 2020; Snowling & Hulme, 2011; Yang et al., 2022). The International Dyslexia Association (IDA) defines dyslexia as a specific learning disability characterized by difficulties with word reading, and/or spelling that involve accuracy, speed, or both, reflecting difficulties in the development of efficient word recognition skills (International Dyslexia Association, 2025). Informed by a 2025 Delphi consensus panel (Carroll et al., 2025), dyslexia has also been described as “a set of processing difficulties affecting reading and spelling, most commonly phonological in nature, but also influenced by working memory, processing speed, and orthographic skills.” This perspective aligns with growing recognition that, although dyslexia is primarily associated with word-level reading and spelling difficulties, its effects may extend to broader language-related processes which limit both decoding efficiency and the development of higher-level oral language skills needed to develop vocabulary growth, syntax development, and discourse organization (Cabbage et al., 2016, 2018; Petersen et al., 2015; Scarborough & Brady, 2002; Share, 2021; Snowling et al., 2003). In fact, approximately 50% of children with dyslexia meet the criteria for developmental language disorder (Bishop et al., 2017; McArthur et al., 2000; Snowling et al., 2019, 2020), and even those children with dyslexia who do not have a diagnosed language disorder remain at risk for language-related weaknesses due to the reciprocal relationship between word recognition, reading comprehension, and oral language comprehension (Cunningham & Stanovich, 1997). This interdependence suggests that phonological deficits not only impair decoding but also limit a child’s ability to acquire and use complex, academic language.

## The reciprocal relationship between reading and language

Academic language refers to the specialized discourse, complex syntax, and vocabulary required for understanding and producing written and spoken language in educational settings (Barnes et al., 2016; Nagy & Townsend, 2012; Schleppegrell, 2001). Much of the written language that students encounter in school, as well as the complexity of the language they are expected to produce in writing, demands a high level of academic language proficiency. Exposure to complex written language improves complex oral language, which in turn impacts a student’s ability to comprehend and engage with complex texts (Cunningham & Stanovich, 1997; Duff et al., 2015; Huettig et al., 2018; Sparks et al., 2014). Given that word recognition, reading, and language skills develop in an interdependent manner, difficulties in any one area can have cascading effects on overall literacy development (Cunningham & Stanovich, 1997; Huettig et al., 2018; Mol & Bus, 2011; Sparks et al., 2014). Children with strong phonological awareness skills are better able to decode unfamiliar words, which facilitates fluent reading and enhances vocabulary and syntactic knowledge. In turn, exposure to text fosters reading comprehension and contributes to further vocabulary and language growth (Beck et al., 1982; Cunningham & Stanovich, 1997; Sparks et al., 2014). A meta-analysis by Mol and Bus (2011) revealed that print exposure has a strong correlation with oral language, accounting for 13% of the variance in oral language skills in primary school and 34% in college. Snowling et al. (2019) found that students with dyslexia, even those without diagnosed DLD, often struggle with complex academic language. These weaknesses may not always meet the threshold for DLD, but still significantly affect a student’s ability to meet expectations for reading comprehension and academic performance. The strong reciprocal relationship among word recognition, reading, and language suggests that most children with dyslexia are likely to exhibit some degree of complex, academic language weakness. In fact, several studies have noted that children with dyslexia often exhibit language limitations similar to their peers with DLD (Snowling & Melby-Lervåg, 2016), particularly in the areas of discourse, syntax, and vocabulary complexity (Adlof et al., 2017; Bishop et al., 2009; Carroll et al., 2014; Duff et al., 2015; Ramus et al., 2013; Rispens & Been, 2007; Robertson & Joannise, 2010; Robertson et al., 2013; Scarborough, 1990; Shankweiler et al., 1995; Snowling et al., 2007; Snowling et al., 2019; Snowling et al., 2020; Vandewalle et al., 2012).

## Identifying language weaknesses using narrative retell

Often, norm-referenced language assessments are utilized to identify language abilities in children with dyslexia (`Eisenmajer et al., 2005; Fraser et al., 2010; Snowling et al., 2019; Snowling et al., 2020). These assessments usually result in children being categorized as either having or not having a language disorder based on specific norms. While these measures can be useful for diagnosing DLD, they have potentially led to an underestimation of the true breadth, variability, and functionality of language competencies among children with dyslexia. Such assessments are often not designed to capture the nuanced and complex language skills that are crucial for academic success (Everatt et al., 2008).

The diverse language profiles observed in children with dyslexia require a more sophisticated approach to assessment, one that not only identifies deficits and strengths but also measures how well students meet curriculum-based language expectations. It is possible that children with dyslexia, including those who do not have DLD, exhibit a higher prevalence of weaker language skills, especially in areas of complex academic language that is often acquired through reading (Duff et al., 2015).

Employing language sample analysis could offer a more nuanced and comprehensive assessment of language abilities in children with dyslexia. In particular, narrative retell analysis can enrich the evaluation framework by providing detailed information on a child’s capacity to understand and produce complex academic language (Fisher et al., 2019a, b) at the discourse (e.g., story grammar), sentence (e.g., subordinate clauses), and vocabulary levels (e.g., complex vocabulary; Costanza-Smith, 2010; LARRC, 2015). Oral narrative language is strongly correlated with broader reading comprehension skills, a relationship underscored by findings that difficulties in narrative production often parallel challenges in understanding text (Miller et al., 2006; Vandewalle et al., 2012). Such correlations promote the inclusion of narrative assessments in language evaluations for children with dyslexia, as they not only highlight underlying language issues but also align closely with narrative-based pedagogical practices. Given that much of academic learning is delivered through narrative-based instruction, evaluating a child’s ability to process and produce narratives can provide valuable insight into their broader language and literacy development and help determine whether a student is meeting curriculum expectations. The benchmarks set by the Common Core State Standards (CCSS) for oral narrative skills provide a valuable metric for assessing whether students are achieving expected educational standards (National Governors Association Center for Best Practices & Council of Chief State School Officers, 2010).

Our research utilizes narrative retell analysis to delve deeper into how children with dyslexia understand and produce complex language structures. By systematically examining narrative retell language samples, we aim to describe a more comprehensive picture of the academic language profiles of children with dyslexia. Therefore, the purpose of this study was to investigate the extent to which school-age students with dyslexia understand and use complex academic language at the discourse, sentence, and vocabulary level. The following research questions were addressed:

1. What percentage of students with dyslexia are below benchmark curriculum expectations for narrative discourse complexity, sentence complexity, and vocabulary complexity for first through sixth grade?
2. What is the percentage of students in first through sixth grade with dyslexia who do not have DLD who are below benchmark curriculum expectations for narrative discourse, sentence, and vocabulary complexity?
3. Is there a significant difference between grade level for students who have dyslexia across narrative discourse, sentence, and vocabulary complexity?
4. Is there a significant difference between students who have dyslexia with DLD and students with dyslexia without DLD across narrative discourse, sentence, and vocabulary complexity by grade level?

## Method

### Participants

The participants included 183 monolingual English-speaking students with dyslexia in first through sixth grade from 13 schools across nine states within the United States. Students were excluded from the study if their primary language was not English or if they did not have dyslexia as an identified disorder. Dyslexia was diagnosed by school psychologists trained in the evaluation of specific learning disabilities. Of the 183 students with dyslexia, 95 attended public school, and of those 95 public school students, 36 (38%) had DLD as confirmed by an Individualized Education Program (IEP) for language services and the *Dynamic Measures of Narrative Language and Decoding* (Petersen et al., 2024). The mean standard score from the *DYMOND* Language Learning Index for the 36 students in the DLD category was 65.53 (SD = 11.44) and the mean standard score for the 59 students in the no DLD category was 94.31 (SD = 14.58). The DYMOND has strong evidence of validity, including for children who are culturally and linguistically diverse, with sensitivity ranging from 91% to 100% and specificity ranging from 89% to 98%. The 88 students who did not attend public school were enrolled in private schools that focused on dyslexia remediation and were not evaluated for eligibility for an IEP nor were they administered the *DYMOND*. All the students in the private schools received tier-1 and tier-2 language support in consultation with a speech-language pathologist (SLP). Demographic information, including race/ethnicity, sex, grade, DLD, and eligibility for free or reduced lunch, used as a proxy measure for socio-economic status, was collected for each participant when available (Table 1).

**Table 1:** Demographic characteristics of participants (n = 183)

Characteristic	*n*	%
Ethnicity		
White	114	62.3%
Black	35	19.1%
Hispanic	4	2.2%
Asian	7	3.8%
Native American/ Alaska Native	9	4.9%
Other	14	7.7%
Sex		
Female	89	48.6%
Male	94	51.4%
Grade		
First	39	21.3%
Second	84	45.9%
Third	31	16.9%
Fourth	11	6.0%
Fifth	8	4.4%
Sixth	10	5.5%
Language Disorder		
Yes	36	19.7%
No	59	32.2%
Undisclosed/undiagnosed	88	48.1%
Free/Reduced Lunch		
Yes	63	34.4%
No	84	45.9%
Undisclosed	36	19.7%

## Procedures

The Institutional Review Board (IRB) from the University of Wyoming approved the study. The Narrative Language Measures-Listening (NLM-L) subtest from the CUBED-3 assessment (Petersen & Spencer, 2023a; Spencer et al., 2023b) was administered to each student by an SLP or a trained research assistant to obtain a language sample. Students were instructed to listen to a grade-level narrative and were then asked to retell the story with as much detail as possible. Each student’s narrative retell was scored by the examiner for inclusion of story grammar elements (narrative discourse structure), adverbial subordinating conjunctions and relative pronouns (sentence complexity), and tier-2 words (vocabulary complexity). Scoring was completed in real-time and then scored again by referencing the student’s transcript, which was generated using artificial intelligence (AI; Microsoft Whisper). The reliability of WhisperAI was previously examined by Konishi-Therkildsen & Petersen (2023), who noted that it transcribed NLM-derived language samples with near-human accuracy (96%), with excellent reliability for overall discourse (ICC ≥ 0.92), sentence complexity (ICC = 0.87), and vocabulary complexity (ICC = 0.83). There was no significant difference in reliability when WhisperAI was required to transcribe language samples from children with language disorder or under less-than-optimal conditions with considerable background noise. Model stories increased in complexity as grade level increased, yet even first grade model narratives included a complete episode with the character, setting, problem, feeling, plan, attempt, consequence, and ending. Second through sixth grade model stories included two complete episodes. All model stories also included numerous adverbial and relative subordinate clauses, and multiple tier-2 vocabulary words. Story grammar elements were scored on a 0–2 point scale, where 1 point was awarded for the inclusion of an incomplete or unclear story grammar element, and 2 points were awarded for complete and clear elements. Sentence complexity and vocabulary complexity scores reflected how frequently complex sentences and complex vocabulary words were used in the narrative retell, receiving one point per use.

## Curriculum expectations

The NLM-L was carefully designed to align with grade level expectations in the CCSS, offering specific cut points that help educators identify students at risk across multiple key areas, including narrative discourse, sentence complexity, and vocabulary complexity. The CCSS emphasize the importance of developing strong reading and writing skills across various content areas and grade levels, specifying that students must demonstrate a clear understanding and effective use of narrative structure, complex sentences, and advanced vocabulary. For narrative discourse, the CCSS require students to engage with texts that provide not only entertainment but also insightful analyses of complex characters and events, necessitating a sophisticated understanding of narrative elements.

For sentence complexity, the standards highlight the ability to construct and interpret compound and complex sentences using a variety of clauses and conjunctions, a critical skill for advanced reading comprehension. Vocabulary standards under the CCSS focus on the understanding and application of academic and domain-specific words and phrases, pushing students to expand and apply their vocabulary knowledge in diverse and meaningful contexts.

The NLM-L’s assessment framework is built to mirror these educational benchmarks, providing educators with a clear metric for evaluating student performance against curriculum expectations. By integrating these standards into the assessment process, the NLM-L helps bridge the gap between evaluation and instruction, offering a targeted approach to language development that is both systematic and aligned with national educational goals. Referencing a large (*N* ~ 5000) national sample, students scoring below the 16th percentile of the CUBED retell composite were classified as high risk. Students below benchmark yet at or above the 16th percentile were classified as moderate risk. Consistent with conventions in literacy and language assessment research, the 16th percentile (approximately one standard deviation below the mean) was selected as the high risk cut point. This threshold has been empirically supported as an optimal balance between sensitivity and specificity for identifying students likely to experience persistent reading or language difficulties (Compton et al., 2006; Fuchs et al., 2004; Jenkins et al., 2007). For a comprehensive overview of how the NLM-L aligns with specific CCSS benchmarks across grade levels, refer to Appendix A, which details the standards and the corresponding assessment criteria used in the NLM-L.

### Training

All examiners participated in an hour-long training on the NLM-L administration and scoring procedures from the first author. During the training, examiners practiced how to administer and score the narrative retell in real-time. Additionally, examiners had access to practice examples prior to administering the assessment to students. Previous research indicated that inter-rater reliability for real-time NLM-L scoring exceeds 90% accuracy (Petersen et al., 2020), and transcript-based scoring achieved approximately 95% accuracy (Petersen & Spencer, 2012). In addition, the reliability of the AI-generated transcript has also been documented at 96% accuracy compared to scoring from a transcription (Petersen & Spencer, 2023a).

## Results

For research question 1, we calculated the means and standard deviations for narrative discourse complexity, sentence complexity, and vocabulary complexity for the entire sample of children with dyslexia (with and without DLD) across grades and evaluated these against established grade-level expectations.

### Narrative discourse complexity

**Fig. 1:**
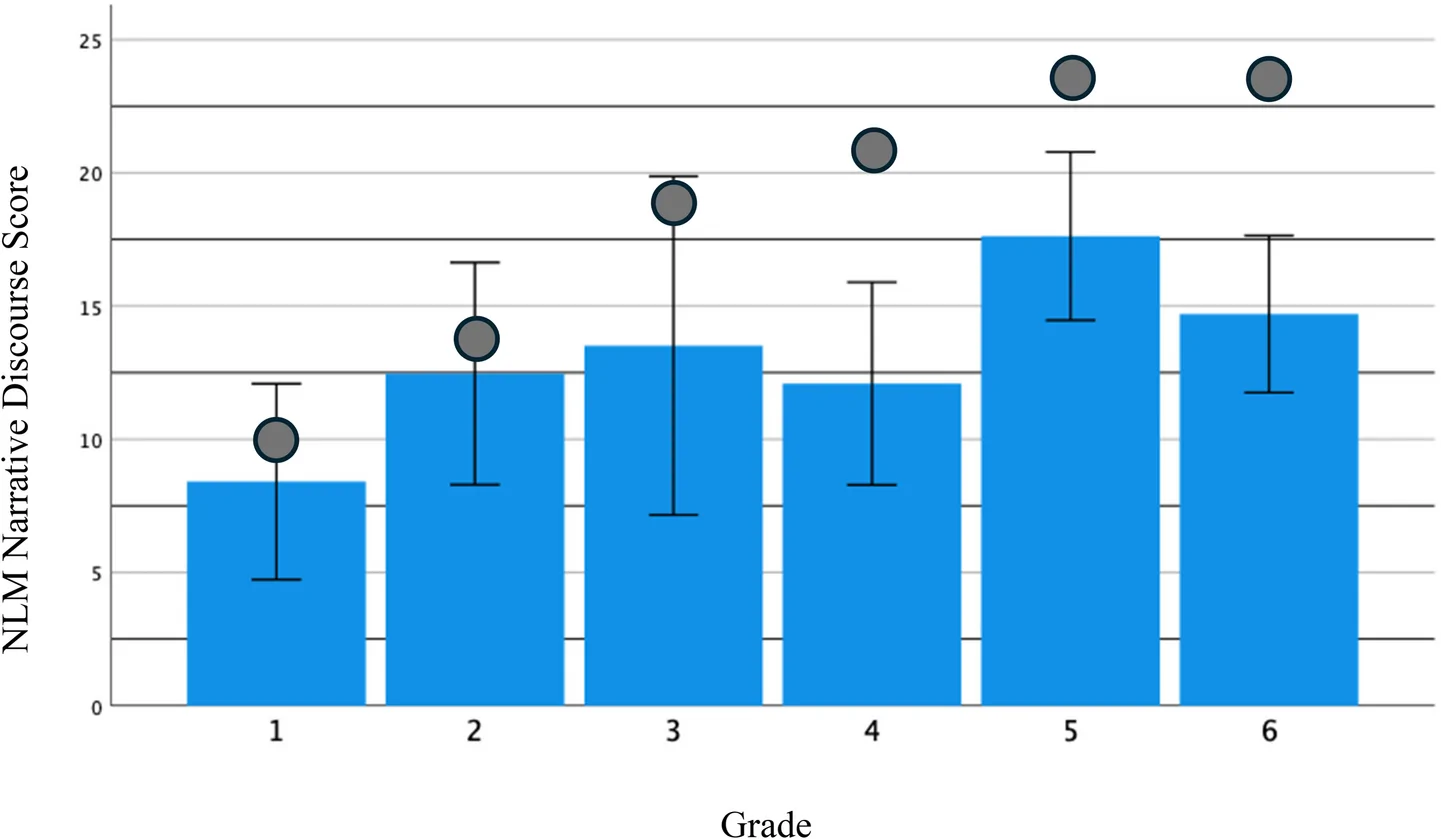
Means and curriculum expectations for narrative discourse complexity for all first through sixth grade students with dyslexia. *Note*. NLM Narrative Discourse is the sum of story grammar elements scored on a scale of 0-2. Error bars are 1 standard deviation. Dots represent end of year NLM benchmark expectations which are aligned with widely adopted curriculum standards

As presented in Table 2 and in Fig. 1, the results indicated that 56.4% of first graders, 54.8% of second graders, 80.6% of third graders, and 100% of students in fourth through sixth grade failed to meet grade-level expectations for discourse complexity.

**Table 2:** Narrative discourse complexity means, standard deviations, benchmark expectations, and percent below expectation by grade for all students with dyslexia

Grade	*n*	Mean	Standard Deviation	Benchmark Expectation	Percent Below Expectation
First	39	8.41	3.68	10	56.40%
Second	84	12.46	4.17	14	54.80%
Third	31	13.52	6.36	19	80.60%
Fourth	11	12.09	3.81	21	100%
Fifth	8	17.63	3.16	23	100%
Sixth	10	14.70	2.95	23	100%

### Sentence complexity

**Fig. 2:**
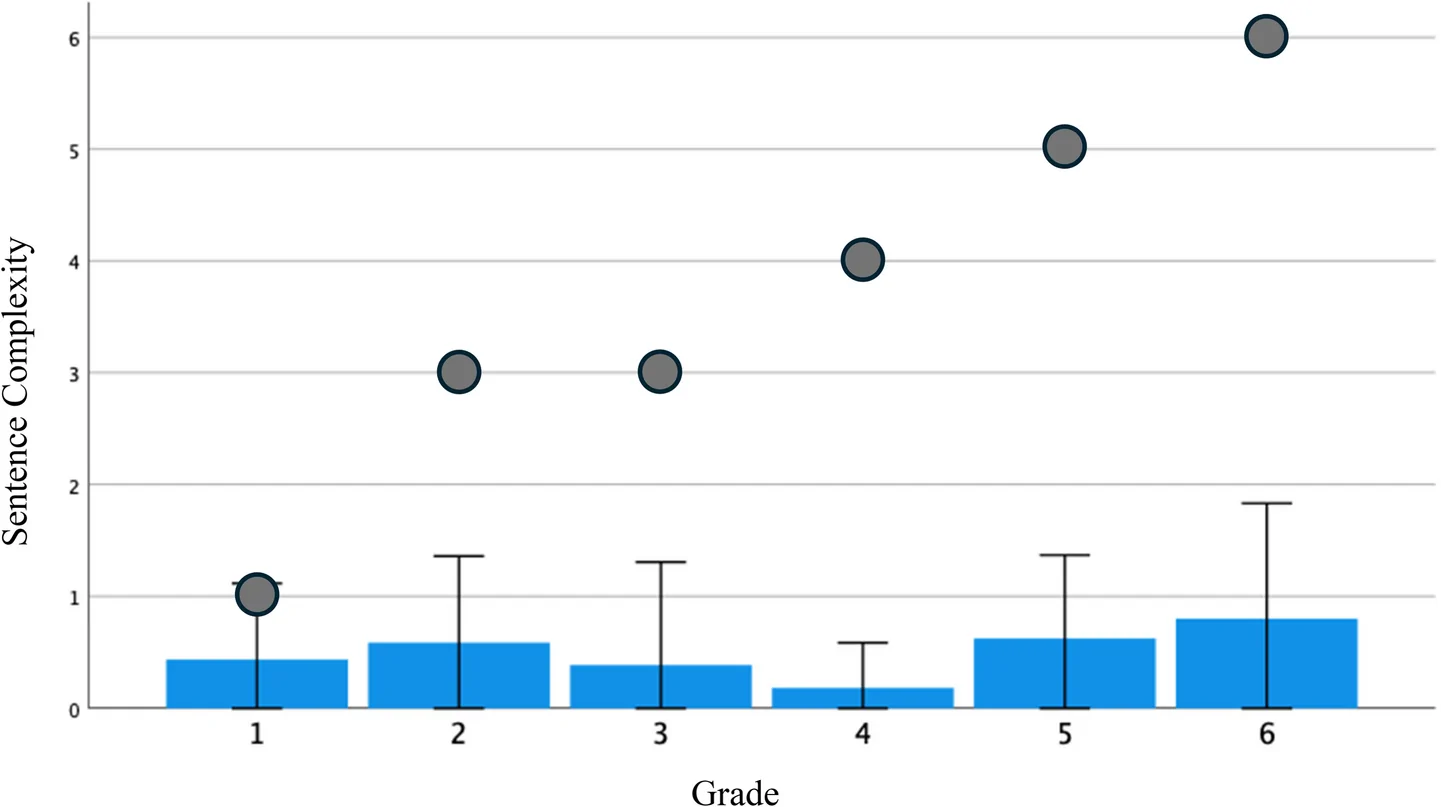
Means and curriculum expectations for sentence complexity for all first through sixth grade students with dyslexia. *Note*. Sentence Complexity is the sum of all adverbial subordinating conjunctions and relative pronouns produced in the narrative retell. Error bars are 1 standard deviation. Dots represent end of year NLM benchmark expectations which are aligned with widely adopted curriculum standards

Further analysis within the same cohort revealed that the majority of students also struggled to meet curriculum expectations for sentence complexity. We analyzed sentence complexity by calculating the means and standard deviations across each grade and comparing those data to benchmark expectations. The results, displayed in Table 3; Fig. 2, indicate that 94.9% of first graders, 100% of second graders, 93.5% of third graders, and 100% of fourth, fifth, and sixth graders did not meet curriculum expectations for sentence complexity. Data were available for 58 of the 84 s-grade students; scores from one private school were unavailable due to a technical recording error that prevented accurate transcription and scoring. Although this reduced the second-grade sample size, representation across other schools and classrooms remained sufficient to interpret overall trends in sentence complexity performance.

**Table 3:** Sentence complexity means, standard deviations, benchmark expectations, and percent below expectation by grade for all students with dyslexia

Grade	*n*	Mean	Standard Deviation	Benchmark Expectation	Percent Below Expectation
First	39	0.44	0.68	1	94.9%
Second	58*	0.59	0.77	3	100%
Third	31	0.39	0.92	3	93.50%
Fourth	11	0.18	0.41	4	100%
Fifth	8	0.63	0.74	5	100%
Sixth	10	0.80	1.03	6	100%

*Sentence complexity scores were available for 58 of the 84 s grade students. Data from one private school could not be analyzed due to a technical recording error

### Vocabulary complexity

**Fig. 3:**
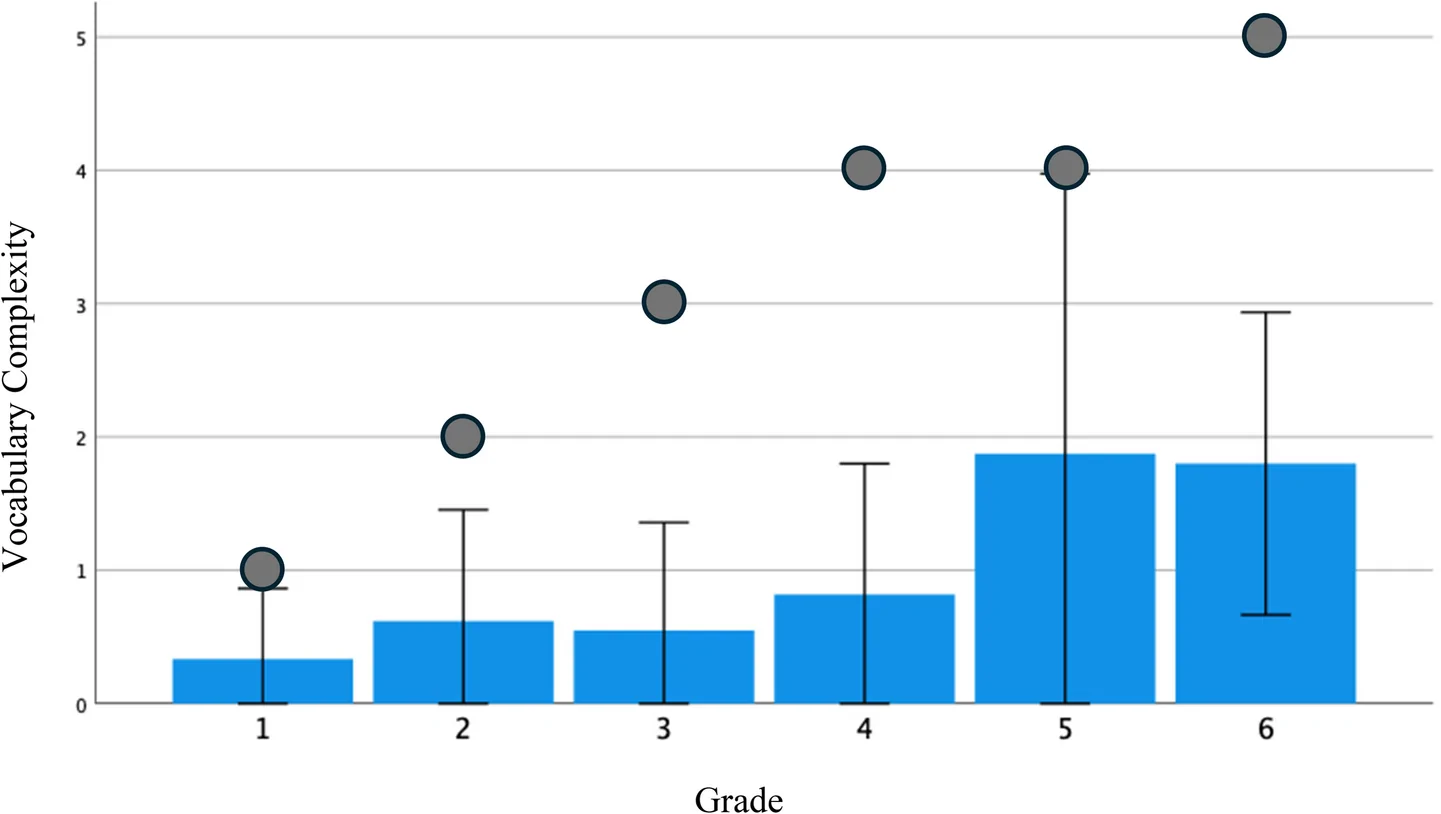
Means and curriculum expectations for vocabulary complexity for all first through sixth grade students with dyslexia. *Note*. Vocabulary Complexity is the sum of all tier-2 vocabulary words produced in the narrative retell. Error bars 1 standard deviation. Dots represent end of year NLM benchmark expectations which are aligned with widely adopted curriculum standards

Vocabulary complexity was analyzed by determining the mean use of tier-2, complex academic vocabulary words for each grade and comparing these means to curriculum expectations. The analysis showed that 97.4% of first graders, 84.5% of second graders, 96.8% of third graders, 100% of students in fourth, 81.8% in fifth grade, and 100% in sixth grades performed below benchmark vocabulary expectations. These findings are presented in Table 4 and visualized in Fig. 3, which highlight the extent of vocabulary limitations across the grades for children with dyslexia.

**Table 4:** Vocabulary complexity means, standard deviations, benchmark expectations, and percent below expectation by grade for all students with dyslexia

Grade	*n*	Mean	Standard Deviation	Benchmark Expectation	Percent Below Expectation
First	39	0.33	0.53	1	97.4%
Second	84	0.62	0.84	2	84.50%
Third	31	0.55	0.81	3	96.80%
Fourth	11	0.82	0.98	4	100%
Fifth	8	1.88	2.10	4	81.80%
Sixth	10	1.80	1.14	5	100%

### Percent of students with dyslexia not meeting grade-level expectations for narrative discourse,sentence, and vocabulary complexity

**Fig. 4:**
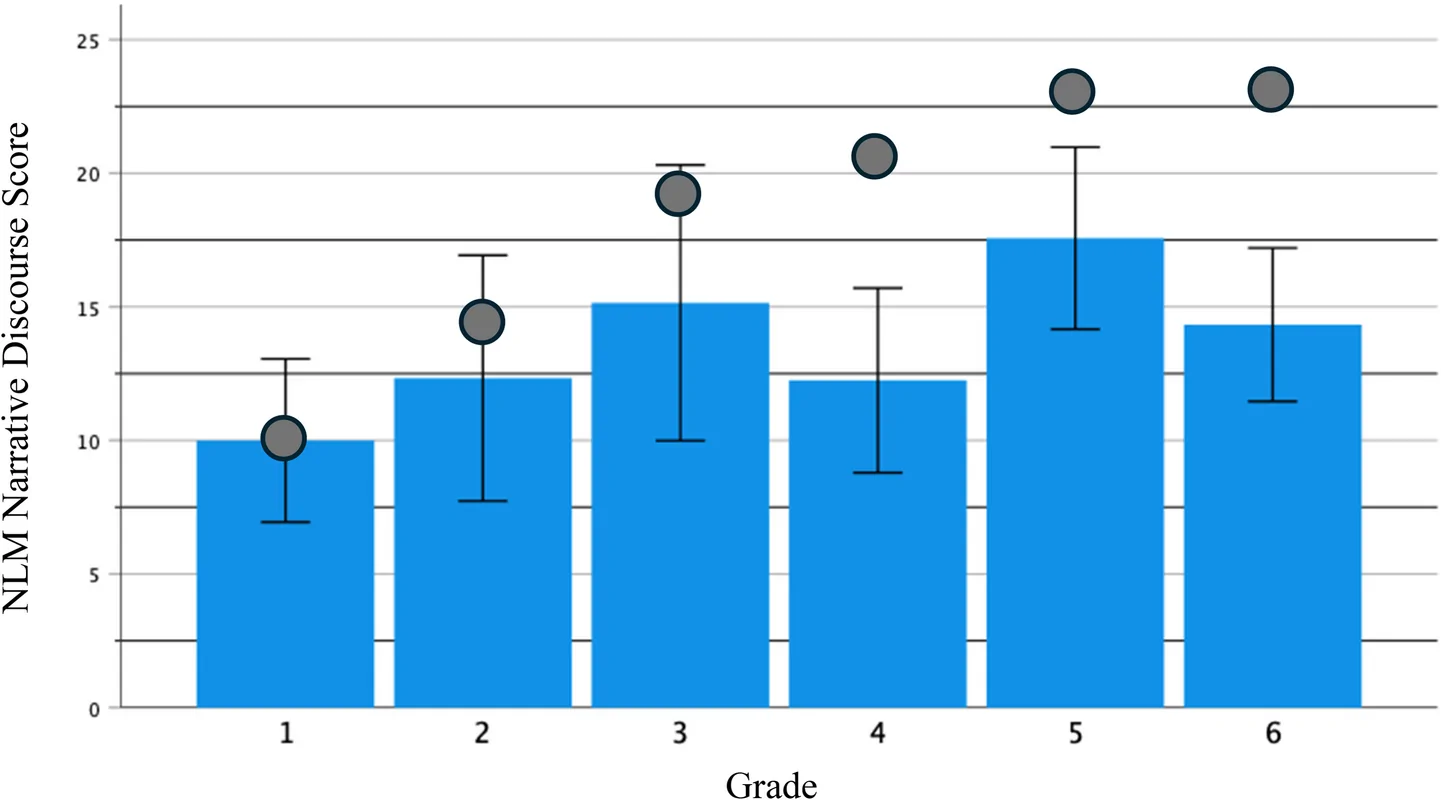
Narrative discourse complexity for children with dyslexia who do not have language disorder. *Note*. NLM Narrative Discourse is the sum of story grammar elements scored on a scale of 0-2. Error bars are 1 standard deviation. Dots represent end of year NLM benchmark expectations which are aligned with widely adopted curriculum standards

**Fig. 5:**
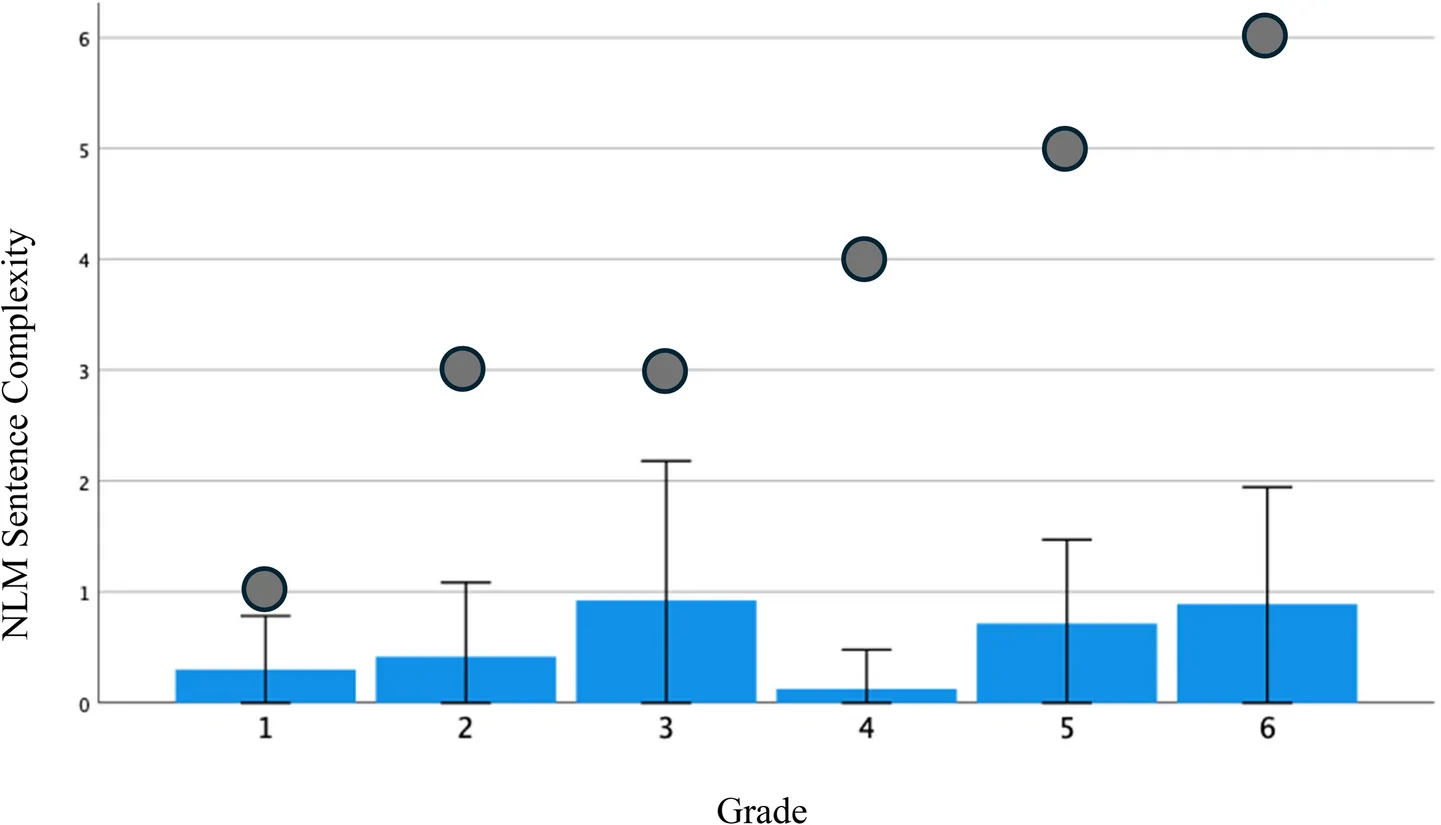
Sentence complexity for children with dyslexia who do not have language disorder. *Note*. Sentence Complexity is the sum of all adverbial subordinating conjunctions and relative pronouns produced in the narrative retell. Error bars are 1 standard deviation. Dots represent end of year NLM benchmark expectations which are aligned with widely adopted curriculum standards

**Fig. 6:**
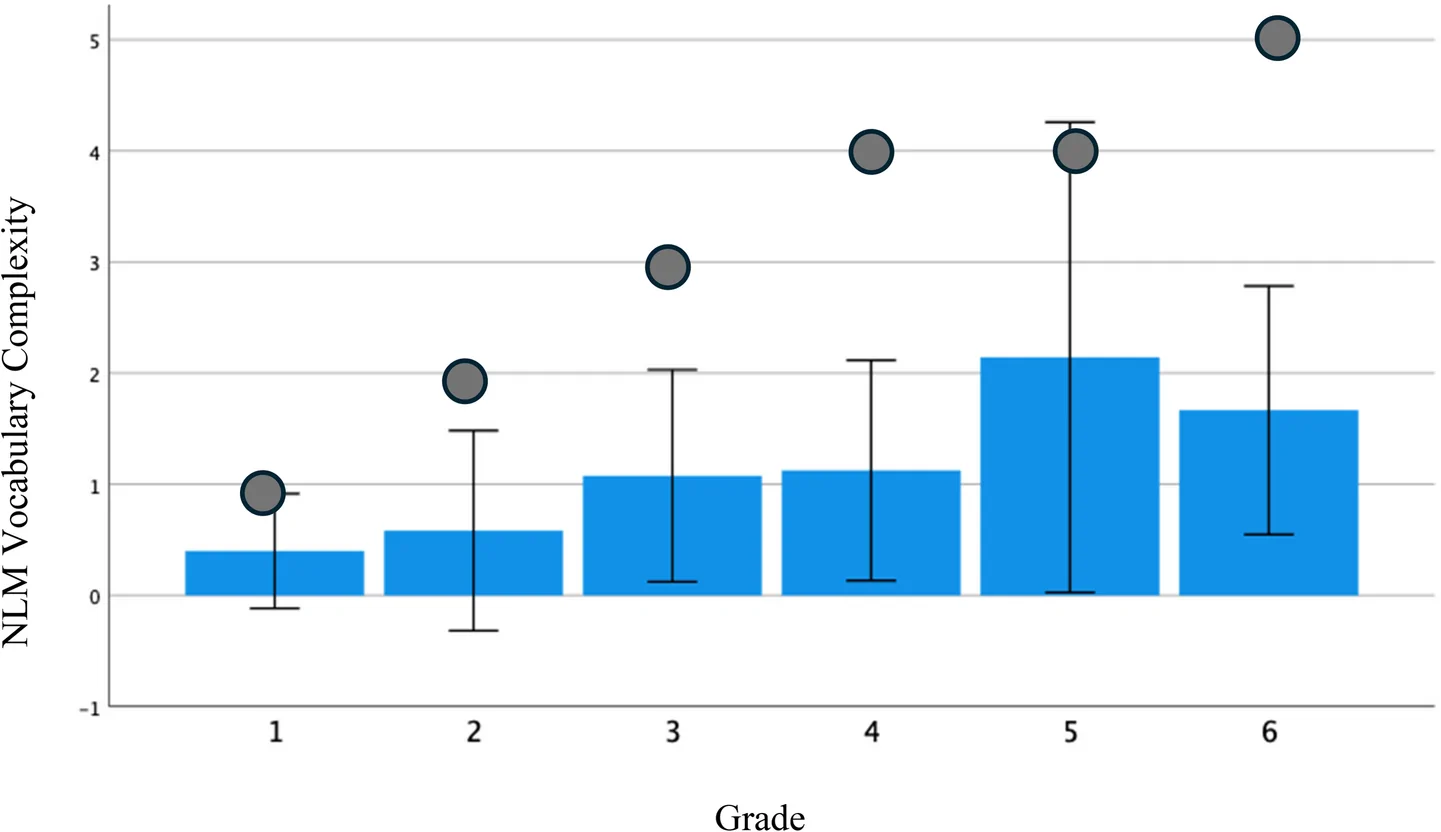
Vocabulary complexity for children with dyslexia who do not have language disorder. *Note*. Vocabulary Complexity is the sum of all tier-2 vocabulary words produced in the narrative retell. Error bars 1 standard deviation. Dots represent end of year NLM benchmark expectations which are aligned with widely adopted curriculum standards

In order to address research question 2, we calculated the means and standard deviations for narrative discourse complexity, sentence complexity, and vocabulary complexity for only the students with dyslexia who did not have DLD across the grades and evaluated these against established grade-level expectations. Tables 5, 6 and 7 display the means, standard deviations, benchmark expectations, and percent below expectations for students with dyslexia who do not have DLD. Figures 4, 5 and 6 display the extent to which these children are meeting benchmark expectations across grades. The majority of children with dyslexia, with the exception of first grade, did not meet curriculum expectations for narrative discourse, sentence complexity, and vocabulary complexity.

**Table 5:** Narrative discourse complexity means, standard deviations, benchmark expectations, and percent below expectations for children with dyslexia with and without language disorder

Grade	*n*	Mean	Standard Deviation	Benchmark Expectation	Percent Below Benchmark Expectation
First					
No DLD	10	10.00	3.06	10	40%
DLD	16	7.19	4.54	10	62.5%
Second					
No DLD	12	12.33	4.60	14	41.7%
DLD	8	7.50	4.57	14	100%
Third					
No DLD	13	15.15	5.16	19	76.9%
DLD	7	5.00	4.62	19	100%
Fourth, Fifth, and Sixth Combined					
No DLD	24	14.58	3.76	< 20	100%
DLD	5	14.20	5.22	< 20	100%

**Table 6:** Sentence complexity means, standard deviations, benchmark expectations, and percent below expectations for children with dyslexia with and without language disorder

Grade	*n*	Mean	Standard Deviation	Benchmark Expectation	Percent Below Benchmark Expectation
First					
No DLD	10	0.30	0.48	1	70%
DLD	16	0.19	0.40	1	81.3%
Second					
No DLD	12	0.42	0.67	3	100%
DLD	8	0.13	0.35	3	100%
Third					
No DLD	13	0.92	1.26	3	84.6%
DLD	7	0.00	0.00	3	100%
Fourth, Fifth, and Sixth Combined					
No DLD	24	0.58	0.83	< 3	100%
DLD	5	0.20	0.45	< 3	100%

**Table 7:** Vocabulary complexity means, standard deviations, benchmark expectations, and percent below expectations for children with dyslexia with and without language disorder

Grade	*n*	Mean	Standard Deviation	Benchmark Expectation	Percent Below Benchmark Expectation
First					
No DLD	10	0.40	0.52	1	60%
DLD	16	0.25	0.45	1	75%
Second					
No DLD	12	0.58	0.90	2	91.7%
DLD	8	0.50	1.07	2	87.5%
Third					
No DLD	13	1.08	0.95	3	92.3%
DLD	7	0.00	0.00	3	100%
Fourth, Fifth, and Sixth Combined					
No DLD	24	1.63	1.44	< 3	100%
DLD	5	0.60	1.34	< 3	100%

### Research questions 3 and 4: descriptive statistics and assumptions

We next analyzed the data to address research questions 3 and 4, examining whether there was a significant difference between grade level for students who have dyslexia and whether there was a significant difference between students who have dyslexia with DLD and students with dyslexia without DLD. Table 5 displays the means and standard deviations for narrative discourse complexity among children diagnosed with dyslexia across different grade levels. Children were also categorized based on the presence or absence of concomitant DLD. The decision to combine grades 4–6 was based on their similar performance levels, relatively consistent curriculum expectations regarding narrative discourse complexity, and considerations regarding statistical power, given the smaller individual sample sizes in these upper grades. Levene’s test for equality of error variances indicated no significant differences in variance between groups, *F*(7, 87) = 0.491, *p* =.491, confirming that the assumption of homogeneity of variances was met for the ANOVA. Table 6 displays the means and standard deviations for sentence complexity. Levene’s Test for Equality of Error Variances indicated significant differences based on the mean (*F*(7, 87) = 4.009, *p* <.001), suggesting a violation of this assumption. However, adjustments based on median and with adjusted degrees of freedom showed non-significant results (*F*(7, 60.847) = 2.084, *p* =.06), suggesting that the assumption may hold under certain conditions. Table 7 displays the means and standard deviations for vocabulary complexity. Levene’s test for equality of error variances showed no significant differences in variance between groups, *F*(7, 87) = 3.607, *p* =.12.

### Differences in grade level: narrative discourse complexity

An Analysis of Variance (ANOVA) was performed to assess the effects of grade level on narrative discourse complexity. The main effect of grade on narrative discourse complexity was significant, *F*(3, 179) = 12.81, *p* <.001, with a partial eta squared of 0.18. Post hoc analyses using the Tukey HSD and two-sided *p* values, indicated there was a significant difference in narrative discourse complexity between first grade and all other grades (*p* <.001) and between second and fourth-sixth grades (*p* =.04). There was no significant difference in narrative discourse complexity between second and third grade (*p* <.27) or between third and fourth-sixth grades (*p* <.39).

### Differences in grade level: sentence complexity

An Analysis of Variance (ANOVA) was performed to assess the effect of grade level on sentence complexity. The main effect was not significant, *F*(3, 153) = 0.54, *p* =.66, with a partial eta squared of 0.01.

### Differences in grade level: vocabulary complexity

The effect of grade on vocabulary complexity was significant, *F*(3, 179) = 9.17, *p* <.001, with a partial eta squared of 0.13. Post hoc comparisons using the Tukey HSD test indicated that students in the combined fourth through sixth grade group produced significantly more complex vocabulary than students in first grade (*p* <.001), second grade (*p* <.001), and third grade (*p* <.001). However, there were no statistically significant differences in vocabulary complexity between first, second, and third grade groups.

### Differences in Language ability: narrative discourse complexity

An ANOVA indicated that there was a main effect, *F*(1, 93) = 31.49, *p* <.001, partial eta squared = 0.25, indicating that language disorder significantly influenced narrative discourse complexity. Pairwise comparisons revealed that there was no significant difference between language ability groups on narrative discourse for first grade (*p* =.097) and between language ability groups on narrative discourse for the combined fourth, fifth, and sixth grade students (*p* =.847). Yet there was a significant difference between language ability groups on narrative discourse for second grade (*p* =.033) and third grade (*p* <.001).

### Differences in Language ability: sentence complexity

An ANOVA revealed that there was a main effect for language disorder, *F*(1, 93) = 8.17, *p* <.01, with a partial eta squared of 0.08. Pairwise comparisons revealed that there was no significant difference between children with and without DLD on sentence complexity for first grade (*p* =.53), second grade (*p* =.28), third grade (*p* =.07), or the combined fourth, fifth, and sixth grades (*p* =.33). Although the main effect of language disorder on sentence complexity was statistically significant, pairwise comparisons within each grade level did not reach significance. This suggests a consistent, though modest, difference across grades rather than a strong effect isolated to any single grade level.

### Differences in Language ability: vocabulary complexity

The main effect of language disorder was significant, *F*(1, 93) = 12.11, *p* <.001, with a partial eta squared of 0.12. Pairwise comparison revealed that there was no significant difference between children with and without DLD on vocabulary complexity for first (*p* =.44), second (*p* =.85), and the combined fourth through sixth grades (*p* =.16), yet there was a significant language difference between children with and without DLD on vocabulary complexity at third grade (*p* <.01).

## Discussion

Our study assessed narrative discourse, sentence, and vocabulary complexities across first through sixth grades in students diagnosed with dyslexia, both with and without DLD. A significant proportion of students with dyslexia did not meet grade-level expectations in these areas. Specifically, challenges in narrative discourse complexity increased from first through sixth grade, culminating in complete non-achievement in grades four to six. Similarly, high levels of difficulty in sentence complexity were consistent across the grades, affecting 94.9% to 100% of students. Vocabulary limitations were significant, with the most severe discrepancies noted in the later grades.

Most students with dyslexia who did not have DLD struggled significantly with benchmark expectations across all language features from second to sixth grade, with the exception of narrative discourse complexity at first grade. Sentence complexity remained relatively unchanged across the grades, indicating that dyslexia impacts the use of adverbial and relative subordination from an early age. Notable improvements in vocabulary complexity were only observed in the upper grades, indicating a delayed developmental trajectory in vocabulary acquisition.

The comparative analysis of dyslexia with and without DLD revealed that the students who did not have DLD did not perform significantly better in narrative discourse than the children with DLD across most grades, although some variations were noted in second and third grades. Students with dyslexia who have DLD may use less adverbial and relative subordinate clauses than their peers with dyslexia who do not have DLD, yet those differences are minimal. Differences in vocabulary complexity were generally minimal, underscoring the widespread challenges associated with vocabulary development among students with dyslexia, regardless of the presence of DLD.

Our results signify that the impact of dyslexia extends well beyond word recognition, affecting critical language skills essential for academic achievement, and that dyslexia can result in language performance that is similar to that of children with DLD. While prior research suggests that approximately 50% of children with dyslexia meet the criteria for DLD (Adlof et al., 2017; Petersen et al., 2018; Bishop et al., 2009, 2017; McArthur et al., 2000; Ramus et al., 2013; Snowling et al., 2019, 2020), this study provides additional evidence that the vast majority of students with dyslexia, from first through sixth grade, exhibit severely limited complex academic language. These language weaknesses, even if subclinical, could drastically hinder reading comprehension and overall academic performance (Catts et al., 2006).

### Discourse complexity and narrative schema

Discourse complexity is essential for students as it involves the ability to use and understand structured language, both spoken and written, in coherent and logically connected ways. This skill underpins narrative comprehension and production, which are critical across all academic subjects. A robust understanding of narrative schema, which includes elements like setting, characters, plot development, conflict, and resolution is pivotal. These elements provide a structural framework that allows students to meaningfully organize and retain textual information.

In this study, discourse complexity was assessed by calculating the mean narrative discourse score from the NLM-L at each grade level. The scoring for story grammar elements was based on the clarity and completeness of their inclusion: two points for a complete and clear inclusion, one point for a partial or unclear inclusion, and zero points for either omitting a story grammar element or including it incorrectly. The analysis revealed that, aside from narrative discourse complexity at first grade, most children with dyslexia who did not present with DLD in grades two through six failed to meet benchmark expectations for narrative discourse. First graders with dyslexia achieved a mean score of 8.41, with over 50% producing fewer than the expected story grammar elements. This suggests that the majority included only a limited number of complete (two-point) or partial (one-point) responses, often omitting or unclearly producing multiple key story grammar elements. Notably, this pattern persisted across the subsequent grade levels. Although some students in first through third grades managed to produce an adequate number of story grammar elements to meet benchmark expectations, 0% of the fourth through sixth graders met these expectations.

There was no significant difference in narrative discourse complexity between second and third grade or between third and fourth-sixth grades. This suggests that once students with dyslexia reach third grade, their narrative discourse complexity may plateau. Furthermore, narrative discourse complexity means were nearly equivalent between the children with and without DLD in older grades. This suggests that by fourth, fifth, and sixth grades, dyslexia’s impact on narrative discourse reaches a level of clinical concern, paralleling the difficulty observed in peers who have both dyslexia and DLD, indicating that the deficits associated with dyslexia alone are profound and become more evident as children progress in school.

These findings highlight the difficulties children with dyslexia experience in both producing and understanding coherent discourse structure, pointing to the broader impact of limited reading experiences and reduced exposure to rich language input. Their struggle with properly structuring narrative elements suggests a significant disconnect from narrative schema, potentially severely hindering their reading comprehension and narrative writing skills. This weakness becomes increasingly consequential as educational demands escalate, particularly when students are expected to analyze literature, synthesize information across various texts, and produce detailed written reports.

### Sentence complexity and the role of subordinate clauses

Sentence complexity is crucial for students as it reflects their ability to construct and comprehend varied sentence structures. Mastery of complex sentences improves academic language both in oral and written communication by enabling students to convey detailed ideas, establish relationships between concepts, and improve overall text cohesion. Additionally, the use of subordinate clauses supports critical thinking and deeper comprehension across academic areas. Sentence complexity plays a key role in reading comprehension as academic texts contain a variety of sentence structures that the student must understand to acquire knowledge from the text. In this study, sentence complexity was measured by calculating the number of adverbial subordinate conjunctions and relative pronouns, which were used as indicators of subordinate clauses. Each use of an adverbial subordinate conjunction or relative pronoun received one point. Our findings indicated a stark deficiency in the ability of students with dyslexia to use complex syntactic forms, with none meeting the minimal expectations for sentence complexity by the fourth grade. Most students did not include a single subordinate clause in their narrative retell – and this is after being asked to retell a story that has multiple subordinate clauses embedded throughout the text. Our results indicated that sentence complexity did not differ between first through sixth-grade students with dyslexia. Students with dyslexia in first grade used a similar number of adverbial and relative subordinate clauses as students with dyslexia in fourth, fifth, and sixth grades. Our results also indicate that students with dyslexia who have DLD may use fewer adverbial and relative subordinate clauses than their peers with dyslexia who do not have DLD, yet those differences are likely minimal. These deficits in sentence complexity not only affect the ability to understand complex sentences in academic texts but also limits the capacity to produce written and oral language that meets educational standards.

Sentence complexity involves the use of varied syntax to construct sentences that convey information efficiently and richly. Complex sentences, particularly those that incorporate subordinate clauses, are vital for expressing for the advanced language tasks required in upper elementary grades and beyond, including explaining cause and effect, expressing temporal sequences, hypothesizing, and providing detailed descriptions. The absence of complex syntax can render their academic discourse simplistic and superficial, which is particularly disadvantageous in subjects like science and history, where complex causal relationships and detailed descriptions are frequently encountered.

### Vocabulary complexity and tier-2 vocabulary

Vocabulary complexity, particularly the use of tier-2 vocabulary, is crucial for academic success. Tier-2 vocabulary consists of high-utility words that are prevalent across academic texts and essential for school success. These words often carry abstract concepts that are critical for understanding and engaging in academic discourse and are central to reading comprehension and writing. The critical role of vocabulary in academic achievement is well-documented, with research indicating that vocabulary development is a strong predictor of reading comprehension and overall academic success (Beck et al., 2002). In this study, we measured vocabulary complexity by calculating the total number of tier-2 vocabulary words the participant used in their story retell.

Students with dyslexia significantly struggled with tier-2 vocabulary across all grade levels. The majority of students did not include any tier-2 vocabulary words in their narrative retell. Our findings indicated that students in the combined fourth through sixth grade group produced significantly more complex vocabulary than students in first, second, and third grade; however, there were no statistically significant differences in vocabulary complexity between first, second, and third grade groups. These results suggest that increases in vocabulary complexity are most pronounced in the upper elementary grades, while early grade levels show comparable performance. Additionally, we found that overall, vocabulary complexity did not vary based on language ability. Children with dyslexia who did and did not have DLD infrequently used tier-2 words in their narrative retells.

### Reading experience and academic language

These findings are consistent with a Matthew effect, in which difficulties in decoding and limited access to print result in deficits across multiple domains of academic language. In the present study, students with dyslexia, regardless of DLD status, demonstrate a significant discrepancy between grade-level benchmark expectations and their performance in discourse, syntax, and vocabulary complexity. The Matthew effect offers an explanation for this pattern, as students with dyslexia face difficulties with reading, which negatively impacts their overall reading experience and, consequently, their exposure to complex discourse, syntax, and vocabulary. Previous research has shown that reading ability predicts the amount of reading students do (Bergen et al., 2018) and that limited reading exposure limits the development of vocabulary and syntactic knowledge (Duff et al., 2015). Furthermore, evidence of a Matthew effect in vocabulary knowledge and sentence and reading comprehension (Röthlisberger et al., 2023), as well as in decoding efficiency, vocabulary, and composite reading scores (Pfost et al., 2014). The current study reinforces these findings by demonstrating that students with dyslexia, regardless of language skills, exhibited a plateau in discourse complexity, minimal growth in syntax complexity, and delayed gains in vocabulary complexity. These developmental patterns likely reflect restricted access to rich academic language input typically acquired through independent reading, further exacerbating the Matthew effect.

### Implications

The findings from our study underscore significant challenges in discourse, sentence, and vocabulary complexities faced by students with dyslexia, signaling the urgent need for a comprehensive reevaluation of educational strategies targeted at this group. While interventions have historically concentrated on phonological and decoding skills, crucial elements that address part of the students’ needs, our research demonstrates that effective support for students with dyslexia must also robustly target the development of rich discourse, complex syntax, and varied vocabulary.

### Integrating language instruction

Educators must incorporate targeted language instruction into the daily curriculum for students with dyslexia (Adlof, 2020). This integration should include structured language interventions that focus on developing coherent and complex narrative structures and enhancing students’ abilities to organize and express their thoughts logically. Such interventions are vital for teaching students to construct detailed narrative frameworks, aiding their understanding and production of narrative discourse structure.

Moreover, there is a crucial need for explicit instruction on complex syntactic forms. Mastery of diverse sentence structures is essential for students to comprehend and convey advanced academic concepts. Vocabulary enrichment through direct instruction, coupled with extensive exposure to a broad range of challenging texts, is equally important. This comprehensive approach not only aids in word recognition but also enhances students’ ability to use these words effectively within various contexts, significantly benefiting their reading comprehension and overall academic performance.

### Empirical evidence of intervention efficacy

The efficacy of contextualized narrative language interventions is well-documented through empirical research demonstrating significant language gains in students with and without disabilities (Gillam et al., 2023, 2024; LARRC, 2014, 2015, 2016; Petersen, 2011; Pico et al., 2021; Ukrainetz & Peterson, 2023). This narrative language intervention research consistently shows improvements across narrative discourse, syntactic complexity, and vocabulary.

Studies using the NLM-L curriculum-aligned outcome measures have shown that benchmark expectations are achievable and can be surpassed, after relatively brief doses of structured literacy intervention for language. For example, Petersen et al. (2022) documented significant enhancements in narrative discourse among kindergarteners after just 14 weeks of narrative instruction, with post-intervention scores on the NLM-L averaging 14.08, reaching levels expected of second graders.

Spencer et al., (2014) observed similar gains in preschoolers following narrative intervention sessions, with post-intervention narrative discourse scores increasing by 8 to 12 points, elevating their performance to match second and third-grade expectations. Additional empirical evidence includes significant vocabulary growth through narrative intervention, as shown in studies by Adlof et al. (2014a, b; Armon-Lotem et al. (2021); Gillam et al. (2014), and Spencer et al. (2019, 2020, 2024) Furthermore, Kirby (2022) noted substantial progress in narrative skills, with first-grade students achieving a mean narrative retell score of 17.83 (benchmark of 10) after receiving targeted interventions. Petersen et al., (2020) found very large effect sizes with second graders after two large group 20-minute sessions per week over eight weeks. Spencer et al. (2014) investigated the effect of narrative language intervention on school-age students with autism, finding that kindergarten, second, and third grade students increased their sentence complexity to a level that meets curriculum expectations. Petersen et al., (2018) found that narrative instruction not only improved oral narrative skills but also significantly boosted writing outcomes, with first graders’ writing scores increasing to an average of 15 on the NLM-L, surpassing second-grade benchmark expectations.

These studies collectively illustrate the potential of multi-component, contextualized structured literacy interventions for language to help students meet and exceed curriculum expectations across various ages and abilities. This robust body of research supports the integration of these interventions into educational frameworks, ensuring that all students, including those with disabilities such as dyslexia, are equipped for academic success and long-term communicative competence.

### The necessity for Language screening and assessment

There is a critical need for comprehensive language screening and assessment to effectively tailor interventions to the individual needs of students with dyslexia (Adlof, 2020). Early and accurate identification of language deficits allows for targeted interventions that can significantly mitigate the long-term academic challenges these students face. Consistent tracking and assessment of language development are vital to ensure that interventions meet the evolving needs of students and are adjusted accordingly to maximize their effectiveness.

Incorporating advanced language instruction into interventions for dyslexia and ensuring rigorous assessment and adjustment of these strategies are crucial for addressing the broad spectrum of language deficits in students with dyslexia. By adopting a holistic approach that includes detailed language instruction and by integrating these strategies into the broader educational framework, educators can significantly enhance the academic outcomes for students with dyslexia. This comprehensive approach ensures that students are equipped with the necessary skills to navigate the complexities of academic language, ultimately enabling them to meet and exceed educational expectations.

### Limitations

This study, while providing insightful findings on the language deficits in students with dyslexia, does have certain limitations that must be acknowledged to contextualize its results accurately. First, the relatively small sample sizes, especially in the higher grades, might limit the robustness and generalizability of our findings. Small cohorts may not fully represent the diverse range of experiences and abilities of students with dyslexia across different educational contexts. Additionally, the exclusive use of the NLM-L as our assessment tool poses another limitation. While the NLM-L is a robust tool for evaluating narrative skills, it may not capture all relevant dimensions of language ability, such as informal conversational skills or the full range of syntactic and vocabulary performance. It is also important to note that only the participants from the public schools were formally identified as having DLD using an IEP and the DYMOND as a reference standard. It is unclear what percentage of students in the private schools had DLD – yet it is clear that the vast majority of those students, including those in the public schools, had considerable weaknesses in producing and understanding complex academic language.

To address these limitations, future research should aim to include a broader array of assessment tools that measure various language domains to provide a more comprehensive understanding of language deficits. Moreover, expanding the participant pool to include a larger and more diverse group of students would help to enhance the generalizability of the findings. This would allow for a richer analysis that could better inform educational practices and intervention strategies, making them more adaptable to a variety of learning environments and student needs.

## Conclusion

The findings from this study call for a significant paradigm shift in how educational interventions for dyslexia are conceptualized and implemented. The pervasive language deficits identified across key areas such as discourse, sentence complexity, and vocabulary underscore the necessity of integrating comprehensive language assessment and instruction into the educational curricula for students with dyslexia. By incorporating supports for enhancing complex language skills, educators can more effectively address the needs of these students. Integrating comprehensive language instruction promises not only to enhance the immediate academic outcomes for students with dyslexia but also equips them with essential linguistic tools for lifelong success. This approach facilitates a deeper engagement with both oral and written language, enabling students to navigate academic challenges more effectively and to participate more fully in society. Ultimately, by fostering a more inclusive and supportive educational environment, we can ensure that students with dyslexia achieve their full potential, both academically and beyond.

The transformation called for by these findings involves both systemic changes in educational practice and a reevaluation of the curriculum to ensure that it meets the diverse needs of all students, especially those with dyslexia. As we continue to uncover the complexities of dyslexia and its impact on language development, it is imperative that our approaches to assessment and intervention evolve correspondingly, ensuring that every student has the tools and opportunities to succeed.
