# Correction: Examining complex academic language in children with dyslexia: a comparative analysis with curriculum standards

**DOI:** 10.1007/s11881-026-00362-5

**Published:** 2026-05-20

**Authors:** Douglas B. Petersen, Gwendolyn H. Stout, Alisa Konishi-Therkildsen, Norma Hancock, Camryn Lettich, Tiffany Hogan

**Affiliations:** 1 Department of Communication Sciences and Disorders, Baylor University, One Bear Place #97332, Waco, TX 76798 United States; 2 Baylor University, Waco, United States; 3 University of Alaska Anchorage, Anchorage, United States; 4 MGH Institute of Health Professions, Boston, United States

**Correction: Annals of Dyslexia**

**https://doi.org/10.1007/s11881-025-00357-8**

In this article Camryn Lettich was missing from the author list.

The original article has been corrected.

